# Dynamic interactions of carbon trading, green certificate trading, and electricity markets: Insights from system dynamics modeling

**DOI:** 10.1371/journal.pone.0304478

**Published:** 2024-06-13

**Authors:** Wei Zhang, Chao Ji, Yongwei Liu, Yuxing Hao, Yang Song, Youxia Cao, Hui Qi

**Affiliations:** 1 Anhui Power Exchange Center Company Limited, Hefei, China; 2 Hunan Power Exchange Center Company Limited, Changsha, China; Shanghai University of Electric Power, CHINA

## Abstract

In the context of the evolving landscape of reduction in carbon emissions and integration of renewable energy, this study uses system dynamics (SD) modeling to explore the interconnected dynamics of carbon trading (CT), tradable green certificate (TGC) trading, and electricity markets. Using differential equations with time delays, the study provides a comprehensive analysis of structural relationships and feedback mechanisms within and between these markets. Key findings reveal the intricate interplay between carbon prices, green certificate prices, and electricity prices under various coupling mechanisms. For example, under the three-market coupling mechanism, carbon trading prices stabilize around 150 Yuan/ton, while green certificate prices reach a peak of 0.45 Yuan/KWH, impacting electricity prices, which fluctuate between 0.33 and 1.09 Yuan / KWH during the simulation period. These quantitative results shed light on nuanced fluctuations in market prices and the dynamics of anticipated purchases and sales volumes within each market. The insights gleaned from this study offer valuable implications for policy makers and market stakeholders in navigating the complexities of carbon emission reduction strategies, the integration of renewable energy and market equilibrium. By understanding the dynamics of multi-market coupling, stakeholders can better formulate policies and strategies to achieve sustainable energy transitions and mitigate impacts of climate change.

## 1. Introduction

The exacerbation of natural disasters due to the current greenhouse effect poses a significant challenge to achieve stable and sustainable development in human society [1]. Globally, issues such as energy depletion and environmental pollution have become unavoidable challenges, necessitating a focus on energy conservation, emissions reduction, and the promotion of green development [2]. With the rapid advancement of new energy generation technologies, the establishment of novel electricity systems has become a crucial infrastructure to ensure the coordinated and stable development of electricity markets [3]. Electricity serves not only as a foundation industry for national economies, but also as a vital component of public utilities [4].

Currently, thermal power generation dominates the electricity industry and is the main source of carbon dioxide emissions [5]. To accomplish the substantial task of reducing carbon dioxide and promote socioeconomic sustainable development, substantial efforts towards the development of renewable energy generation industries are imperative for China’s power sector to transition towards low-carbon development [6]. At the end of 2023, the newly added electricity generation capacity reached 330 million kilowatts, with a total installed capacity of 2.9 billion kilowatts, indicating a stable development in the electricity industry [7]. Renewable energy accounted for a total installed capacity of 1.45 billion kilowatts, exceeding fossil fuel-based power generation capacity, which represents a significant achievement in China’s transition to green and low-carbon energy transition [8]. Renewable energy generation reached 3 trillion kilowatt hours, representing one third of the total electricity consumption [9].

The rapid expansion of renewable energy and the reduction in the proportion of non-clean energy sources have elevated the level of green and low-carbon practices in the energy industry [10]. However, due to the inherent uncertainty associated with the utilization of renewable energy, the high penetration of new energy sources into the electricity market can cause significant fluctuations, reducing the overall utilization efficiency of renewable energy [11]. In the process of achieving the carbon peak and carbon neutrality goals, maximizing the use of electricity generated from renewable sources poses a significant challenge [12]. Addressing issues such as grid connection difficulties and the idle capacity of wind and solar energy facilities, China has proposed implementing a renewable energy quota system in its "14th Five-Year Plan," with the aim of actively promoting the development of renewable energy generation industries [13]. Furthermore, the introduction of a renewable energy quota system by the State Council, as outlined in the "2023 Report on the Development of China’s Strategic Emerging Industries", aims to support the full coverage of renewable energy generation through guaranteed purchase mechanisms [14]. The renewable energy quota system requires that a certain proportion of the electricity produced by power generation companies must come from green sources, decoupling the environmental and social benefits derived from the generation of renewable energy from the electricity itself and facilitating competition within the renewable energy sector [15].

Carbon trading (CT) mechanisms involve government-mandated regulations within specific regions and time frames, restricting carbon emissions from power generation activities to prescribed levels [16]. Climate change mitigation institutions distribute free carbon emissions quotas to coal-fired power plant operators, allowing for trading of excess emissions among them. This system aims to restrict carbon emissions through market mechanisms, encouraging more effective management and reduction of carbon emissions by coal power plants to achieve climate change mitigation objectives [17]. The trading of certified green certificates (TGCs) involves the conversion of renewable energy generation certificates issued by renewable energy generation companies and coal power plants into tradable certificates by renewable energy information management centers [18]. Beyond the prescribed quotas, the trading of green energy beyond the renewable energy quota system and between renewable energy and coal-fired power plants is permitted [19].

Regarding CT research, Wang et al. [20] proposed a novel hybrid approach to predict carbon trading prices, utilizing Bayesian neural networks to construct a data set of price information for model training, offering new insights to predict and evaluate carbon prices. Xian et al. [21] evaluated the synergistic benefits and mechanisms of carbon trading pilot policies on carbon emissions and atmospheric pollutant emissions in the national electricity, industry, transportation, and residential sectors using a time-varying difference model. Their findings indicated that carbon trading policies can help reduce carbon emissions and control air pollution. Hou et al. [22], based on a natural experiment involving carbon trading policies, analyzed data from China from 2004 to 2019 using an intermediary model. They found that carbon trading policies not only directly improve carbon emission efficiency, but also have a synergistic effect on reducing sulfur emissions. Tian et al. [23] employed a panel data fixed-effects model to analyze data from listed companies, examining the impact of digital economic development on carbon emissions from the perspective of the carbon trading market. They concluded that for every unit increase in the level of digital economic development in the carbon trading market, carbon emissions decrease by 0.417 units, contributing to the dynamic development of carbon trading policies. Feng et al. [24] investigated the impact of carbon trading policies on carbon emissions reduction, employing propensity score matching and double difference methods to study the causal relationship between carbon trading policies and carbon emissions reduction. They found that the implementation of carbon trading policies effectively promotes carbon emissions reduction. Wang et al. [25] combined China’s carbon emission quota and verified emission reduction policies, designing a carbon credit trading mechanism that accommodates both electricity and carbon trading, establishing a carbon-electricity coupled market model with the carbon credit trading center as the core, and quantifying the impact of introducing carbon credit constraints on node marginal electricity prices. Gan et al. [26] analyzed the fluctuation trends of fluctuation in China’s macroeconomics and environmental quality before and after the establishment of the carbon market, exploring the impact mechanisms of the carbon market and technological progress on China’s pollution reduction and carbon emission reduction through the construction of models in the environmental sector, models in the carbon emission trading plan, and models of the carbon emission trading mechanism.

As for TGC research, Coria et al. [27] examined the impact of ownership structures of participants in the Swedish TGC system on TGC prices, analyzing detailed TGC transaction-level data to explore differences in TGC prices between cross-ownership companies and non-cross-ownership companies. Fu et al. [28], based on real options theory, investigated a new regional grid pricing mechanism for solar photovoltaic energy under the TGC policy in China, calculating internal returns ranging from 8% to 16%, balancing consumer burdens with investor interests, and improving the competitiveness of photovoltaic energy in China’s energy market. Safarzadeh et al. [29] compared the tradable white certificate market and the TGC market, applying evolutionary game theory to elucidate multi-agent problems in the electricity supply chain, advancing the progress of sustainable energy projects in developing countries. Meng et al. [30] studied TGC negotiations and cooperative operations between multiple entities in the comprehensive energy sector, building a variable TGC limit price model. The results indicated that the proposed model effectively promoted green certificate trading between intelligent agents, with trading volumes increasing by 20.83% compared to noncooperative scenarios.

The study of the mechanisms of the electricity market trading under CT and TGC holds significant theoretical and practical significance [31]. Specifically, it manifests itself in: (1) CT primarily affects high-carbon emitting enterprises on the distribution side of the power grid, where thermal power plants directly influence the layout of the power generation side [32]. Acceptance of CT by power plant operators and their electricity generation decisions under carbon trading will affect the proportion of thermal power integrated into the grid, thus disturbing grid stability and, in certain situations, causing price fluctuations. Therefore, investigating the intervention of CT in the electricity market is imperative [33]. (2) TGC directly impacts the proportion of renewable energy integrated on the power generation side of the grid and collaborates with CT to affect users. TGC not only involves renewable energy generation companies, high-carbon emitting enterprises, and users, but also imposes quota requirements on national renewable energy electricity quotas [34]. Therefore, the decision making and strategic behavior of the market entities in TGC affect the changes in the energy structure of the grid. Understanding how to maximize one’s benefits in a multi-agent game market environment is a crucial issue to be explored in the electricity market [35]. (3) When both CT and TGC simultaneously influence the electricity market, the synergistic effects between the three markets will inevitably affect the existing electricity market [29]. Factors such as power generation and emission reduction within various power generation groups, CT by high carbon emitting enterprises, and emission reductions through TGC are crucial factors that affect both the power generation and consumption sides of the grid [36]. Studying the interrelationships and mutual effects among these markets and, based on this, exploring the issue of equilibrium in market benefits can provide constructive suggestions for market entities.

The development of low-carbon and environmentally friendly electricity trading and the construction of green electricity markets have received widespread attention from scholars. Durante et al. [37], based on extensive research into the European electricity market, studied the interdependencies between electricity prices, demand and renewable energy, building a multivariate Copula model to elucidate daily variations in electricity prices, renewable energy, and demand, offering guidance for the promotion of green energy. For the rapidly developing renewable energy sector, Shimomura et al. [38] proposed a model based on machine learning technology and explainable artificial intelligence, estimating the multidirectional impact of renewable energy on the Japanese electricity market. They found that demand has the greatest contribution to market prices, followed by solar power generation and operable power facility capacities. When demand is high and solar power generation is low, market prices rise, providing insight into variations in renewable energy and other policy factors. Oliveira et al. [39] studied the impact of the electricity market on capacity trading and renewable energy technology, demonstrating that regional investments can effectively lower energy prices and significantly increase investments in renewable technologies. Xu et al. [40] regarded the electricity market, the green certificate market tradable, and the carbon emission trading market as a comprehensive energy platform that connects carbon emissions with electricity, connecting power generation companies and retailers. They proposed more optimal pricing strategies to stimulate renewable energy electricity generation and consumption in the carbon-electricity coupled market.

Although there are many studies on the individual impacts of CT and TGC mechanisms on electricity prices, research on their coupling with the electricity trading market is considered an effective means of addressing the negative externalities of electricity production through marketization. Research on the impacts of TGC and TC on the electricity market and the proposal of practical policy implementation suggestions are of the highest academic and practical importance.

This study constructs system dynamics (SD) models based on specific theoretical frameworks for the CT market, the TGC market, and the coupling of the three markets, assigning values to various variables within each model. By studying the changes in market prices under different models, conducting comprehensive analyses of the impacts of the CT and TGC markets on the electricity market, and conducting mechanistic analyses of the interactions among the three markets, this research calculates the variation curves of electricity prices under the coupling of the three markets, providing theoretical support for promoting the participation of CT and TGC in the Chinese electricity market. This, in turn, advances the green and sustainable development of the electricity market, alleviates the pressure on government subsidies for renewable energy, and enhances the competitiveness of renewable energy in electricity market transactions.

## 2. Model

### 2.1 Model frame

To investigate the interrelated model of carbon trading, green certificate trading, and electricity markets, it is necessary to employ System Dynamics (SD) modeling [41]. The essence of SD models lies in the form of first-order differential equations with time delays, utilized to study changes caused by various factors within and outside a system simultaneously [42]. This involves analyzing observed data and feedback information from the external environment to forecast system behavior. The internal structure and feedback mechanisms of the system collectively determine its dynamic behavioral patterns.

The advantage of SD models lies in their ability to handle high-order, non-linear, time-varying systems with multiple feedback loops, enabling quantitative analysis of the structure and functions of such complex systems. The distinctive feature of SD models is their ability to analyze current issues on a relatively long-time scale, thereby studying engineering problems from a strategic and long-term perspective. Typically, these models comprise three types of equations: state equations, rate equations, and auxiliary equations, each corresponding to three respective variables.

**Fig 1:**
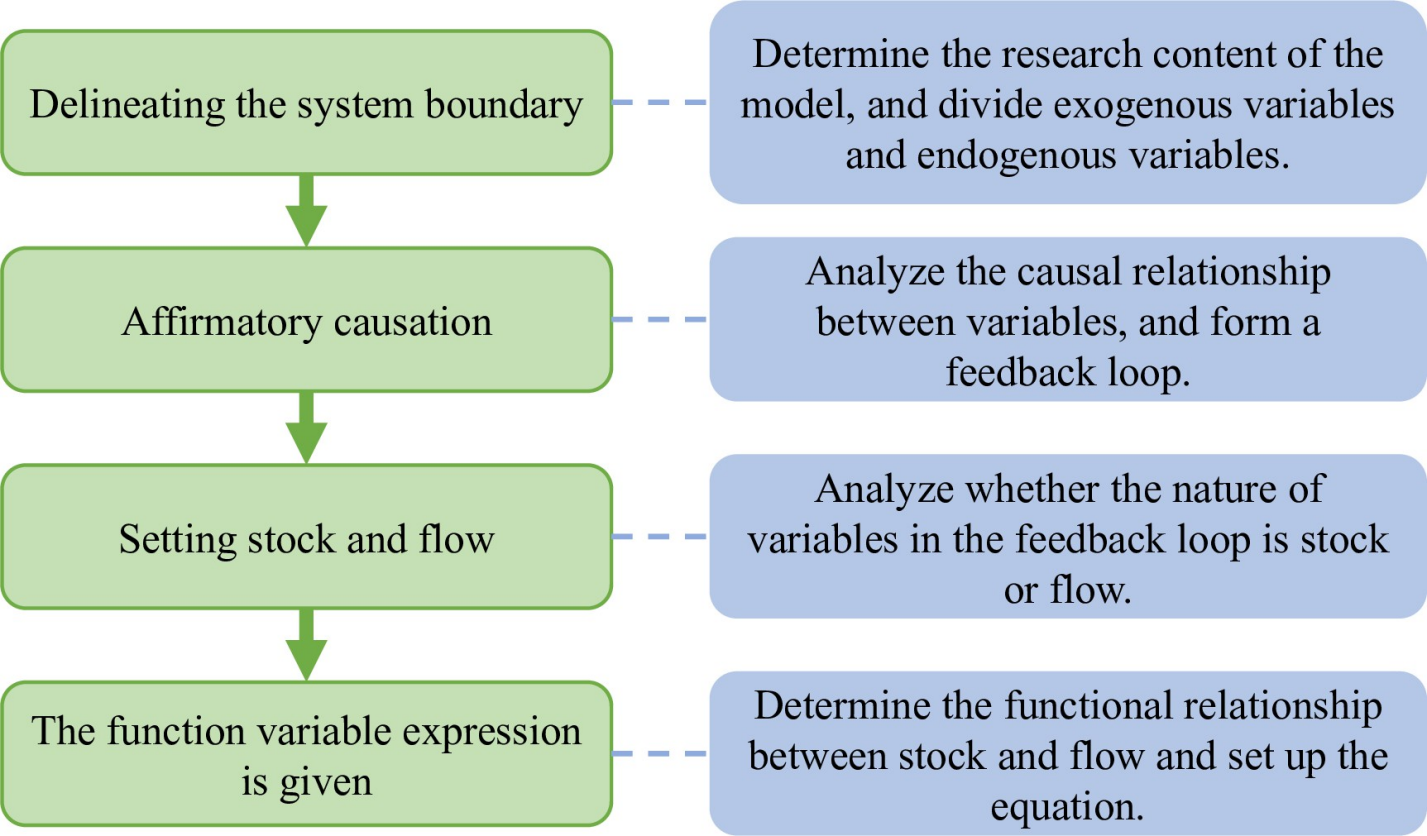
SD model process.

SD models encapsulate two primary causal relationships: positive causality and negative causality, the process of the model shown in Fig 1. The structural relationships of all complex systems are made up of these two causal relationships. Positive causality means that an increase in one variable results in an increase in another, while negative causality indicates that an increase in one variable leads to a decrease in another. For ease of identification and analysis, positive causality is denoted by "+" and negative causality by "-".

The causal relationships between the CT market, the TGC market, and the electricity market ultimately manifest themselves in carbon prices, green certificate prices, and electricity prices. The core of these markets lies in price fluctuations driving market regulation, ultimately balancing supply and demand to maintain stable market operations. Therefore, separate model designs are necessary for the CT market, TGC market, and multi-market.

### 2.2 Model design

In the context of first-order positive feedback systems, their specific manifestation is characterized by monotonically exponential growth. The differential equation representing exponential growth is as follows:

$$\overset{•}{x}=kx,\;k>0$$ (1)

Solution:

$$x(0)=x_{0}e^{kt}=x_{0}e^{\frac{t}{\tau }},\;\tau =\frac{1}{k}$$ (2)

The differential equation can also be expressed as

$$\overset{•}{x}=kx-b,\;k>0$$ (3)

Solution:

$$x(t)=[x(0)-\frac{b}{k}]e^{kt}+\frac{b}{k}$$ (4)

When *b*<0, the system exhibits an exponential growth trend due to factor *x*(0)≥0; when *b* = 0, the equation is $\overset{•}{x}=kx,(k>0)$; however, when *b*>0, there exist three scenarios, and the trend of the system in these scenarios depends on the relative relationship between the values of *x*(0) and $\frac{b}{k}$.

①When $x(0)>\frac{b}{k}$, $[x(0)-\frac{b}{k}]>0$ shows an exponential growth trend;
②When $x(0)=\frac{b}{k}$, $[x(0)-\frac{b}{k}]=0$ is a constant and its value is always equal to $\frac{b}{k}$;
③When $x(0)<\frac{b}{k}$, $[x(0)-\frac{b}{k}]<0$ shows an exponential collapse trend.

The first-order positive feedback system exhibits a singular development trajectory, where the introduction of an external variable at the initial value results in monotonous variation and asymptotic numerical growth. Such a curve lacks convergence and extremal values. To ensure that the system operates within realistic boundaries, the incorporation of negative feedback is essential.

The differential equation for first-order negative feedback is as follows.


$$\overset{•}{x}=-kx+b,\;k>0$$ (5)

Solution:

$$x(t)=[x(0)-\frac{b}{k}]e^{-kt}+\frac{b}{k}$$ (6)

Ⅰ. When *b* = 0, the curve decays exponentially and the differential equation is:

$$\overset{•}{x}=-kx,k>0$$ (7)

Solution:

$$x(t)=x(0)e^{-kt}$$ (8)

Ⅱ. When *b*<0, is similar to *b* = 0, whose equilibrium point is $\frac{b}{k}$;
Ⅲ. When *b*>0, the curve trend is related to the size of *x*(0), but all three trends converge to $\frac{b}{k}$ and are independent of the initial value.

#### 2.2.1 Coupling model of the CT market and the electricity market

**Fig 2:**
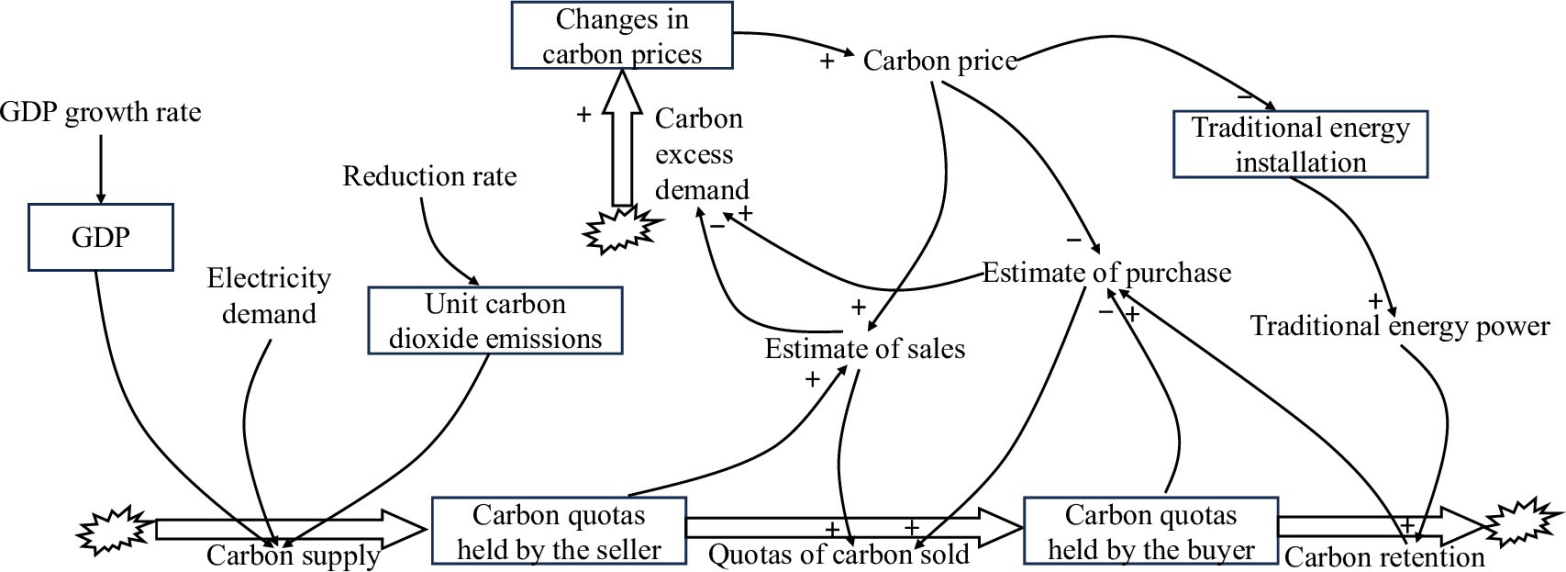
SD model of the CT and electricity market.

The SD model for the CT market and the electricity market is shown in Fig 2.

The production costs of traditional energy generation companies, represented by thermal power plants, are significantly influenced by carbon quotas, as depicted in Fig 2. Insufficient carbon quotas for thermal power companies require purchasing from the CT market, typically through transactions with other entities possessing surplus carbon quotas. In instances where aggregate market demand exceeds total supply, escalating carbon prices challenge thermal power enterprises to offset emissions reduction costs with profits, thereby leading to a reduction in the newly installed capacity for traditional energy generation. On the contrary, a decrease in overall demand within the thermal power sector precipitates a decrease in carbon prices, facilitating the attainment of a relatively stable equilibrium state in both the CT and the electricity markets.

The systemic dynamic models of CT markets and electricity markets consist of three fundamental variables along with exogenous variables and constants. The equations for state variables are as follows:

$$V_{cp}=P_{c0}+E_{c}$$ (9)

$$I_{c}=i_{c0}+\frac{d_{e0}\times r_{e}\times 0.5\times P_{c0}}{12\times P_{c}\times t_{c}}$$ (10)

$$Q_{b}=0+Q_{t}-D_{c}$$ (11)

$$Q_{s}=q_{s0}+S_{c}-Q_{t}$$ (12)

where *V*_*cp*_ is the change in carbon price; *P*_*c*0_ is the initial price of carbon; *E*_*c*_ is the excess demand for carbon; *I*_*c*_ is the installed capacity of traditional energy sources; *i*_*c*0_ is the initial installed capacity of traditional energy sources; *d*_*e*0_ is the initial demand for electricity; *r*_*e*_ is the growth rate of electricity demand; *P*_*c*_ is the price of carbon; *t*_*c*_ is the annual number of hours of use of the installed capacity of traditional energy sources; *Q*_*b*_ is the amount of carbon allowances held by buyers; *Q*_*t*_ is the amount of carbon allowances sold; *D*_*c*_ is the demand for carbon; *Q*_*s*_ is the amount of carbon allowances held by sellers; *q*_*s*0_ is carbon initial allowance and *S*_*c*_ is the supply of carbon.

The equation for the rate variable is:

$$E_{c}=\frac{\left(q_{pc}-q_{sc}\right)}{q_{sc}}$$ (13)

$$S_{c}=\frac{2.43Ge}{10000}$$ (14)

$$D_{c}=\frac{Q_{ec}\times c}{10000}$$ (15)

Where *E*_*c*_ is the excess demand for carbon allowances; *q*_*pc*_ is the projected purchases; *q*_*sc*_ is the projected sales; *S*_*c*_ is the carbon supply; *G* is the GDP; *e* is the share of the power sector; *D*_*c*_ is the carbon demand; *Q*_*ec*_ is the conventional energy power; and *c* is the carbon dioxide emission factor.

The equation for the exogenous variable is as follows:

$$G=\frac{g_{0}+g_{0}\times r_{g}}{12}$$ (16)

$$D_{e}=e\times G$$ (17)

Where, *g*_0_ is the initial value of GDP; *r*_*g*_ is the GDP growth rate; *D*_*e*_ is the electricity demand.

The constant values for the SD models of the CT market and the electricity market are shown in Table 1.

**Table 1:** The constant values for the SD models of the CT market and the electricity market.

Constant	Value
Carbon dioxide emission factor *c*	0.78
The share of the power sector *e*	6%
Electricity demand growth rate *r*_*e*_	6%
GDP growth rate *r*_*g*_	5.2%
Carbon initial allowance *q*_*s0*_	4.08

#### 2.2.2 Coupling model of TGC and electricity market

**Fig 3:**
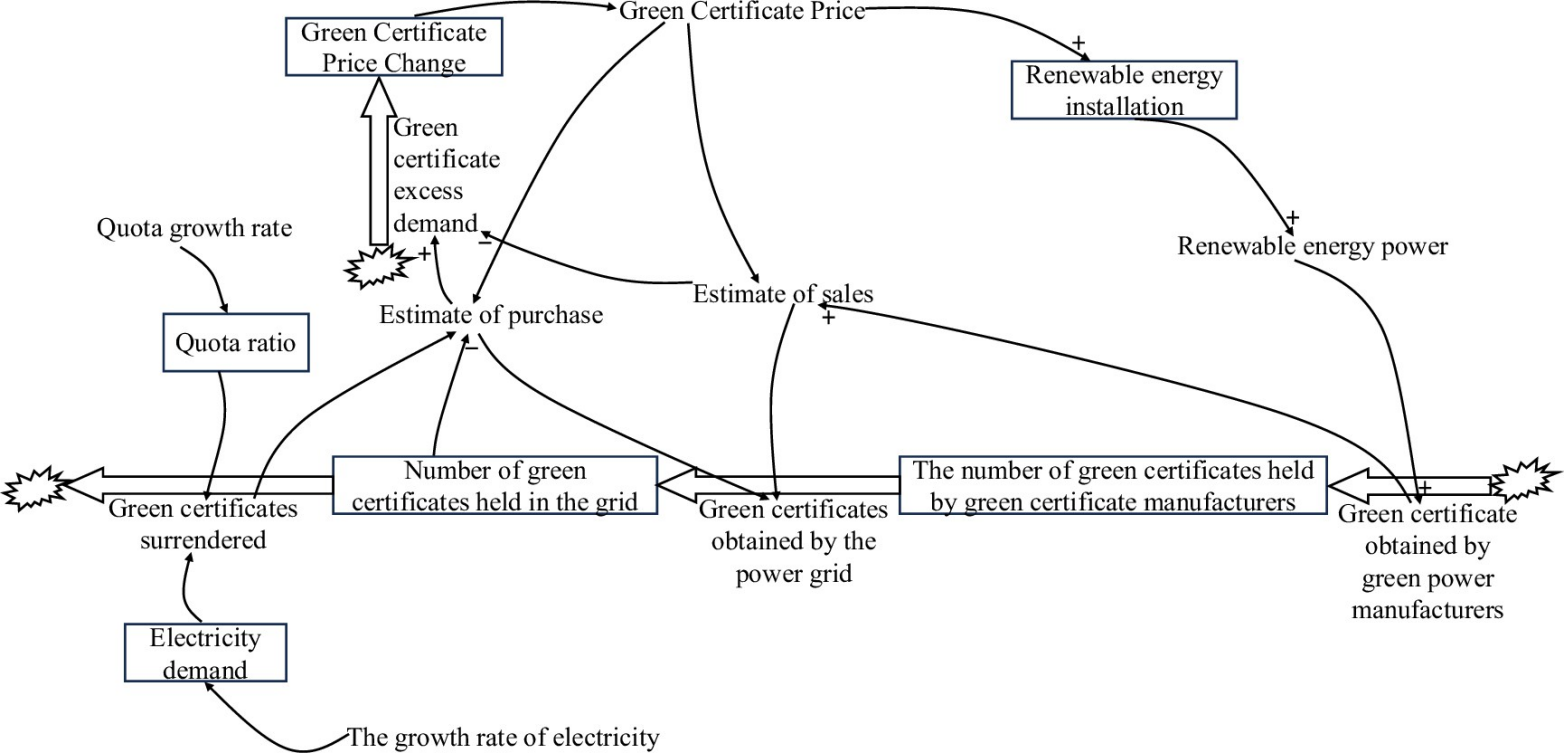
TGC and SD model of the electricity market.

The SD model for the TGC market and the electricity market is shown in Fig 3.

The SD model of the TGC market and the electricity market is based on the variation in the prices of green certificates to achieve negative feedback loops, as shown in Fig 3. The fluctuations in the prices of green certificates are determined by the supply-demand relationship of green certificates. The ratio of anticipated purchases to anticipated sales is expected to determine the trend and magnitude of the next-step change in green certificate prices.

The state variable equation of the TGC market and the electricity market model is as follows:

$$V_{gp}=P_{g0}+E_{g}$$ (18)

$$I_{g}=i_{g0}+\frac{d_{e0}\times r_{e}\times 0.5\times P_{g0}}{12\times P_{g}\times t_{g}}$$ (19)

$$C_{ger}=C_{ger0}+O_{ger}-O_{grid}$$ (20)

$$C_{grid}=0+O_{grid}-O_{up}$$ (21)

Where *V*_*gp*_ is the change in the price of green certificates; *P*_*g*0_ is the initial price of green certificates; *E*_*g*_ is the excess demand for green certificates; *I*_*g*_ is the installed renewable energy capacity; *i*_*g*0_ is the initial installed renewable energy capacity; *P*_*g*_ is the price of green certificates; *t*_*g*_ is the average annual utilisation of hours of renewable energy; *C*_*ger*_ is the green certificates held by green power producers; *C*_*ger*0_ is the initial green certificates held by green power producers; *O*_*ger*_ is the green certificates obtained by green power producers; *O*_*grid*_ is the green certificates obtained by the electricity network; *C*_*grid*_ is the green certificates held by the electricity network; and *O*_*up*_ is the green certificates surrendered.

The equation for the rate variable is:

$$E_{g}=\frac{\left(q_{pg}-q_{sg}\right)}{q_{sg}}$$ (22)

$$Q_{ger}=\frac{Q_{eg}}{1000000}$$ (23)

$$O_{up}=D_{e}\times P_{q}$$ (24)

where *q*_*pg*_ is the projected purchases, *q*_*sg*_ is the projected sales, *Q*_*eg*_ is the amount of renewable electricity, *D*_*e*_ is the demand for electricity, and *P*_*q*_ is the quota share.

The equation of the auxiliary variable is as follows:

$$Q_{eg}=\frac{I_{g}\times t_{g}}{12}$$ (25)

Where *Q*_*eg*_ is the amount of electricity from renewable energy; *I*_*g*_ is the installed renewable energy capacity; and *t*_*g*_ is the average annual utilisation hours of renewable energy.

The equation for the exogenous variable is as follows:

$$D_{e}=\frac{d_{e0}}{12}+\frac{d_{e0}\times r_{e}}{12}$$ (26)

$$P_{q}=P_{q0}+r_{pg}$$ (27)

Where *D*_*e*_ is electricity demand; *d*_*e*0_ is the initial value of electricity demand; *r*_*e*_ is the growth rate of electricity demand; *P*_*q*0_ is the initial value of quota share; and *r*_*pg*_ is the growth rate of quota share.

The constant values for the SD models for the TGC market and the electricity market are shown in Table 2.

**Table 2:** The values of the constants for the SD models of the TGC market and the electricity market.

Constant	Value
Power demand initial value *d*_*e*0_	91500
Electricity demand growth rate *r*_*e*_	6%
Quota ratio initial value *P*_*q*0_	5%
Quota growth rate *r*_*pg*_	0.13%
Average annual hours of renewable energy *t*_*g*_	6644

#### 2.2.3 Multimarket convergence transaction model

**Fig 4:**
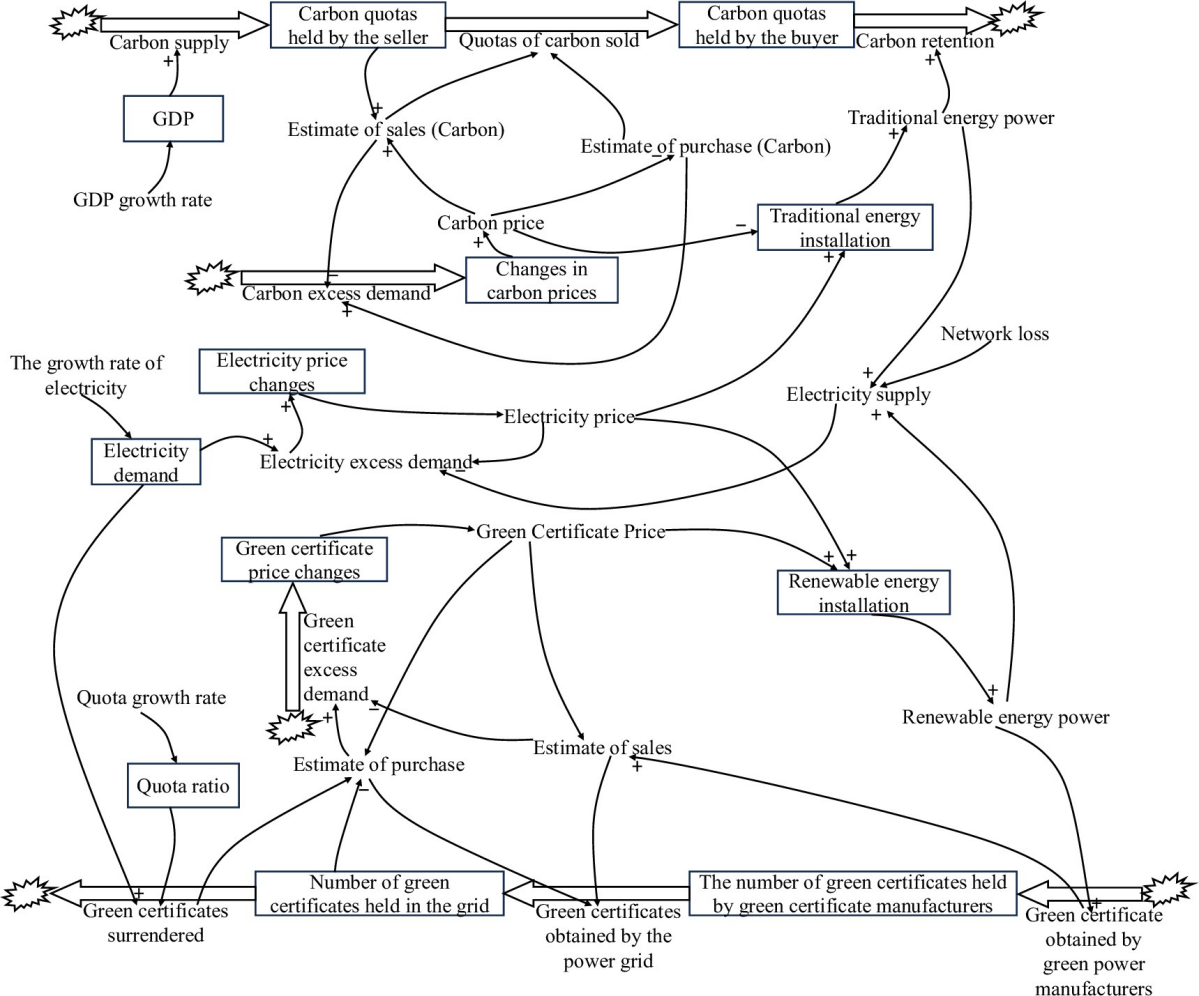
SD model of multiple markets.

The SD model of the tripartite trading market of CT, TGC, and electricity market is shown in Fig 4.

The predominant influence of electricity prices on these three markets is evident, as shown in Fig 4. Acting as a bridge connecting the other two markets, the electricity market facilitates the transmission of external influencing factors from one market to the other two in a chain reaction. CT and TGC markets impact the electricity market through carbon and green certificate prices, respectively. The escalation of carbon prices inhibits the installation and generation capacity of traditional energy sources, while higher prices of green certificates stimulate the generation of renewable energy. The electricity supply in the electricity market is based on both traditional and renewable energy sources. Synergistic interactions among the three markets mutually influence each other, ultimately achieving a balanced state under shared constraints.

The relevant state variable equation for the electricity market is:

$$D_{e}=\frac{d_{e0}}{12}+\frac{d_{e0}\times r_{e}}{12p_{e0}}$$ (28)

$$V_{ep}=p_{e0}+E_{e}$$ (29)

Where *D*_*e*_ is electricity demand; *d*_*e*0_ is the initial value of electricity demand; *r*_*e*_ is the growth rate of electricity demand; *p*_*e*0_ is the initial value of electricity price; *V*_*ep*_ is the change in electricity price; and *E*_*e*_ is the excess demand for electricity.

The equation for the rate variable is:

$$E_{e}=\frac{P_{e}(D_{e}-S_{e})}{50D_{e}}$$ (30)

Where *E*_*e*_ is the excess demand for electricity; *P*_*e*_ is the price of electricity; *D*_*e*_ is electricity demand; and *S*_*e*_ is the supply of electricity.

The equation of the auxiliary variable is as follows:

$$S_{e}=(Q_{ec}+Q_{eg})(1-l)$$ (31)

where *P*_*e*_ is the price of electricity; *S*_*e*_ is the supply of electricity; *Q*_*ec*_ is the amount of electricity from conventional energy sources; *Q*_*eg*_ is the amount of electricity from renewable energy sources, and *l* is the grid loss.

The values of the constants for the SD models of the CT, TGC, and the electricity market are shown in Table 3.

**Table 3:** The values of the constants for the SD models of the CT, TGC and the electricity market.

Constant	Value
Power demand initial value *d*_*e*0_	91500
Electricity price initial value *P*_*e*0_	0.38
Electricity demand growth rate *r*_*e*_	6%
Grid loss *l*	4.6%

## 3. Results and discussions

### 3.1 Coupling analysis of CT market

In the interconnected model of the CT and electricity markets, traditional energy generation enterprises, exemplified by thermal power plants, experience significant influence on production costs due to carbon quota. Insufficient carbon quotas prompt thermal power producers to acquire from the CT market, acquiring quotas through transactions with entities possessing surplus allowances. In scenarios where total market demand exceeds supply, elevated carbon prices challenge thermal power producers in balancing emission reduction costs with profitability, consequently leading to a reduction in newly installed capacity for traditional energy generation.

**Fig 5:**
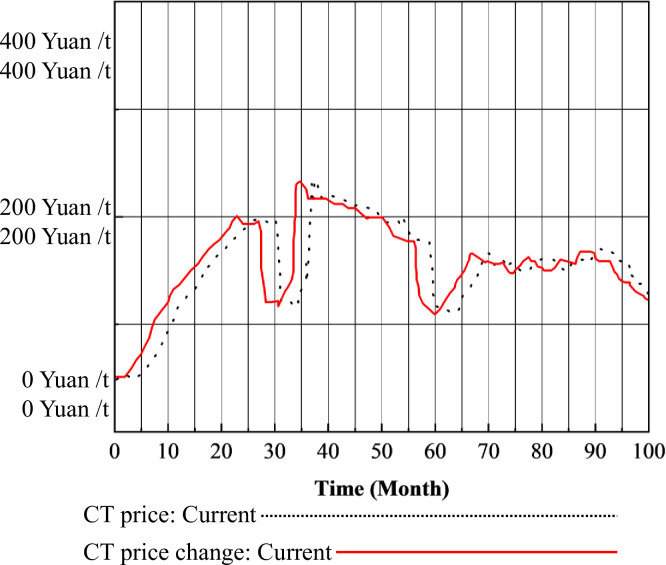
CT prices under the CT mechanism.

**Fig 6:**
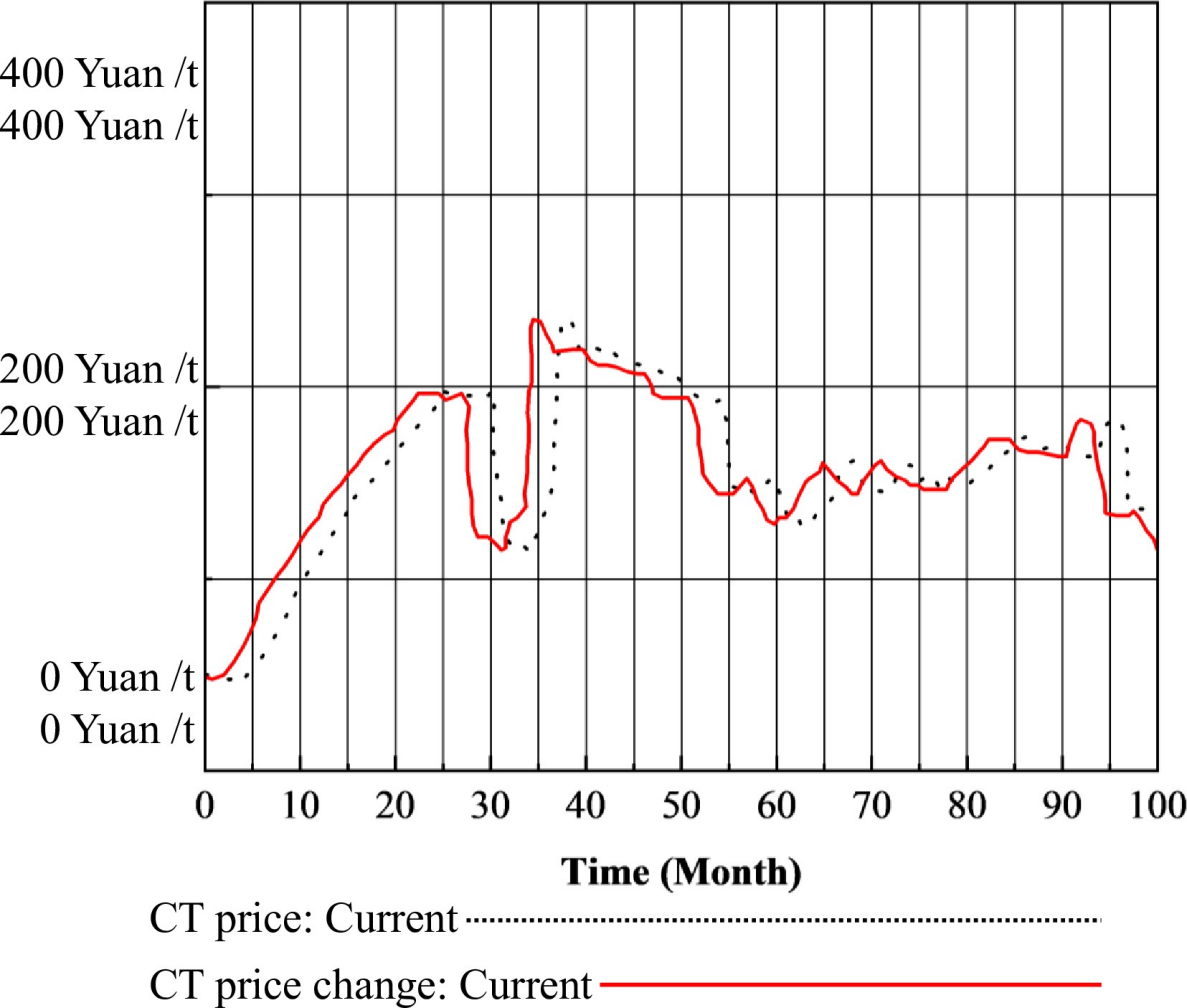
CT prices under the three-market coupling mechanism.

By computing the SD models of the CT market in isolation and within the framework of the mu-market coupling mechanism, CT prices can be determined, as illustrated in Figs 4, 5 and 6. Fig 5 shows the CT prices under the CT mechanism alone, while Fig 6 illustrates the CT prices under the multimarket coupling mechanism.

In Fig 5, the price of CT initially increases from the baseline price to a higher level in the short term, then experiences a minor decline back to the median price. Subsequently, it surges to a higher level and maintains stability for a period before fluctuating to reach a price equilibrium. From months 0 to 10, the CT price remains relatively stable at the baseline price of 50 Yuan/ton. From month 10 to 25, the CT price steadily rises to a higher level of 200 Yuan / tons. However, in month 28, it quickly drops back to the median price of 100 Yuan/ton, then rapidly ascends to its peak at 232.7 yuan per ton in month 34. It remains around 200 Yuan/ton from month 34 to 50, then decreases to the median price of 100 Yuan/ton from month 50 to 60. Subsequently, from month 67 to 90, fluctuations occur, ultimately stabilizing in the vicinity of 150 Yuan/ton, exhibiting minor oscillations.

In Fig 6, it is evident that under the three-market mechanism, CT prices experience an earlier decline compared to the standalone CT mechanism, with no significant signs of large fluctuations. They reach equilibrium more quickly around the 150 Yuan/ton price line.

The reasons for the fluctuation of CT prices in Fig 5 are as follows. I. Baseline phase (months 0–10): Traditional energy generation enterprises already hold a portion of carbon quotas without significant excess demand. Meanwhile, these enterprises are still observing the CT market, thus maintaining CT prices at approximately 50 Yuan/ton. II. Steady-up phase (months 10–25): Following the implementation of carbon emission trading, the first batch of traditional energy installations is completed under the drive of carbon prices, electricity prices, and electricity demand. With stable growth in carbon quota demand, power plants find their quotas insufficient to support their emissions, resulting in a demand-supply gap and CT prices rising to 200 Yuan/ton. Ⅲ. Rapid decline phase (months 25–30): As CT prices increase rapidly, the cost of traditional energy power plants to use excess free carbon dioxide emissions increases. Consequently, the profit margins for these plants diminish. To hedge risks, some power plants invest in renewable energy, slowing the growth rate of traditional energy installations. Economic growth leads to an increase in carbon emissions supply, resulting in a short-term oversupply situation in the market and a rapid decline in CT prices. Ⅳ. Sustained high phase (months 34–50): After experiencing a decline in CT prices, the increase in profit margins due to lower CT prices, coupled with high electricity prices, leads traditional energy generation enterprises to increase investments, accelerating the growth rate of traditional energy installations and significantly increasing CT demand. The slow supply of CT causes CT prices to increase. Ⅴ. Price decline phase (months 50–60): The continued high CT prices compress profit margins for traditional energy generation, prompting a repetition of the process seen in Ⅲ. Ultimately, prices drop to the median of 100 Yuan / tons. Ⅵ. Stability amid fluctuations (months 67–90): Following Ⅳ, investments by traditional energy generation companies become more rational and cautious. CT demand steadily increases, while CT supply gradually meets demand. Under the influence of market mechanisms, the carbon trading market achieves equilibrium. CT prices experience minor fluctuations around the 150 Yuan / tons price line, eventually reaching stability.

**Fig 7:**
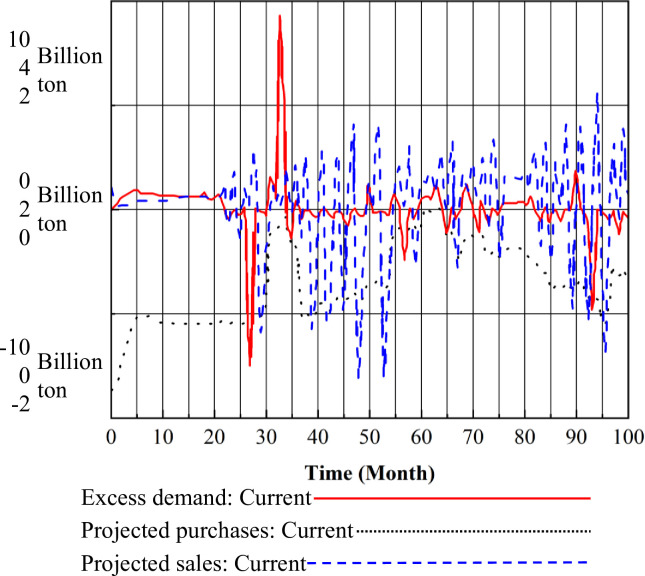
Changes in excess CT demand and related variables.

The trends in the projected purchases of CT by power producers, the projected sales, and the excess demand for CT on the market are shown in Fig 7.

The trends and reasons for the variables in Fig 7 are as follows: Ⅰ. For buyer generators, during the initial phase of carbon emission trading (0–25 months), the CT market experiences a supply-demand imbalance, leading to a gradual increase in CT prices. Consequently, the expected purchase volume of carbon quotas by buyer generators slowly increases, reaching 1 billion tons per month. As the first batch of traditional energy installations is completed and operationalized, traditional energy generation increases, resulting in increased carbon emissions and CT demand. The anticipated volumes of CT also increase accordingly, as shown by the peak at month 24. With a slowdown in traditional energy installation investments, expected purchase volumes initially decline but gradually increase in tandem with changes in installations, ultimately stabilizing as the market reaches stability. Ⅱ. For seller generators, during the initial phase of carbon emission trading (0–25 months), due to the continuous increase in CT prices and a steady rise in national carbon quota supply, power generators do not have surplus carbon quota for significant sales. Consequently, anticipated sales volumes of CT remain around 1.2 billion tons. As CT prices decrease, profits decrease, leading to a reduction in anticipated sales volumes, occasionally intersecting with anticipated purchase volumes in the short term. Ⅲ. Regarding the CT excess demand curve, between months 0 and 20, anticipated purchase volumes consistently exceed anticipated sales volumes, resulting in excess demand greater than 0. In month 22, a significant gap between anticipated purchase and sales volumes causes excess demand to quickly drop to 0. In month 27, with an increase in traditional energy installation capacity, anticipated purchase volumes increase, causing excess demand to rebound to 0. Subsequently, with the gradual stabilization of the carbon trading market and CT prices, along with more rational investment decisions by power generators, excess demand experiences minimal fluctuation, only varying with changes in the difference between anticipated purchase and sales volumes.

### 3.2 Coupling analysis of the TGC market

**Fig 8:**
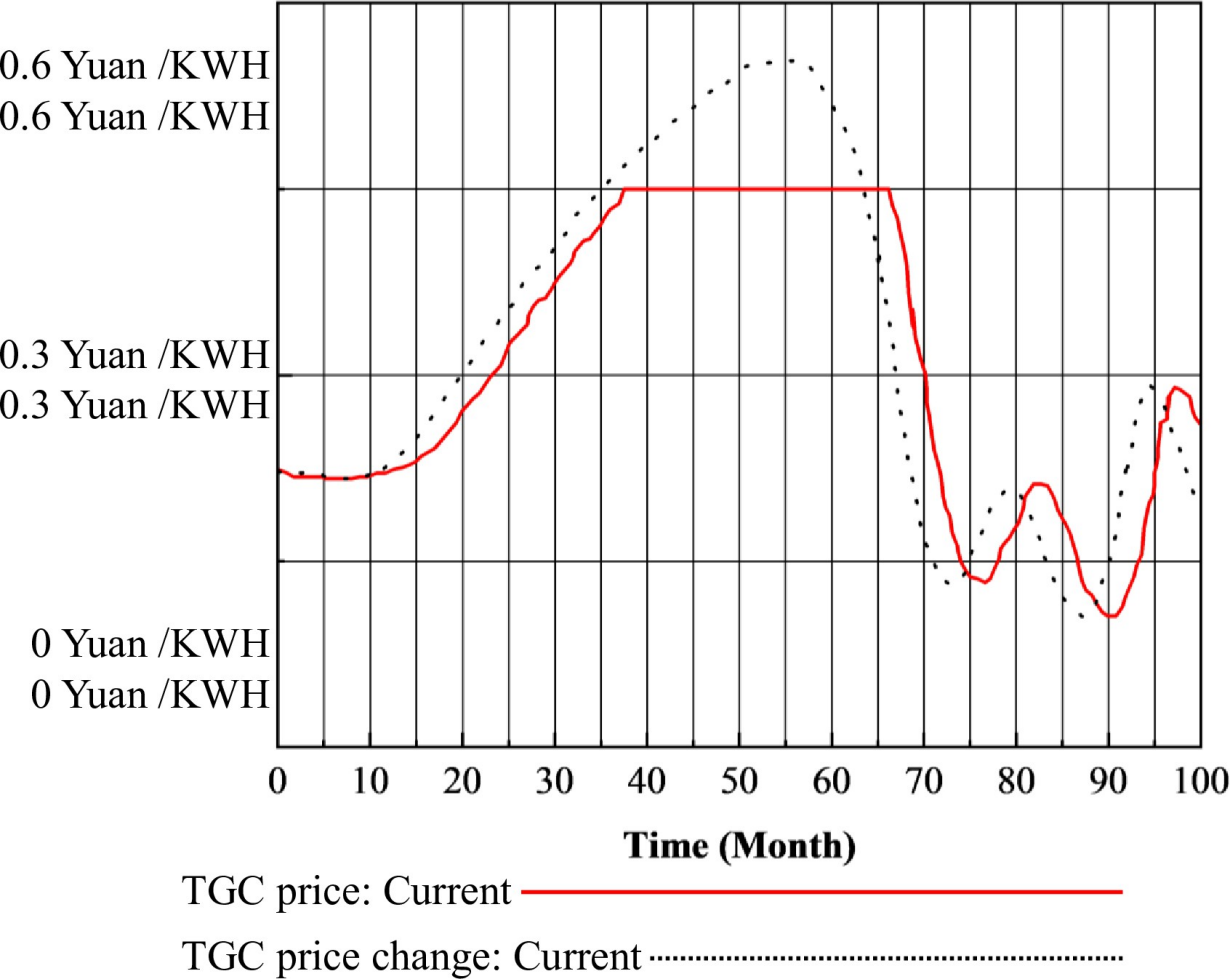
TGC prices under the TGC mechanism.

**Fig 9:**
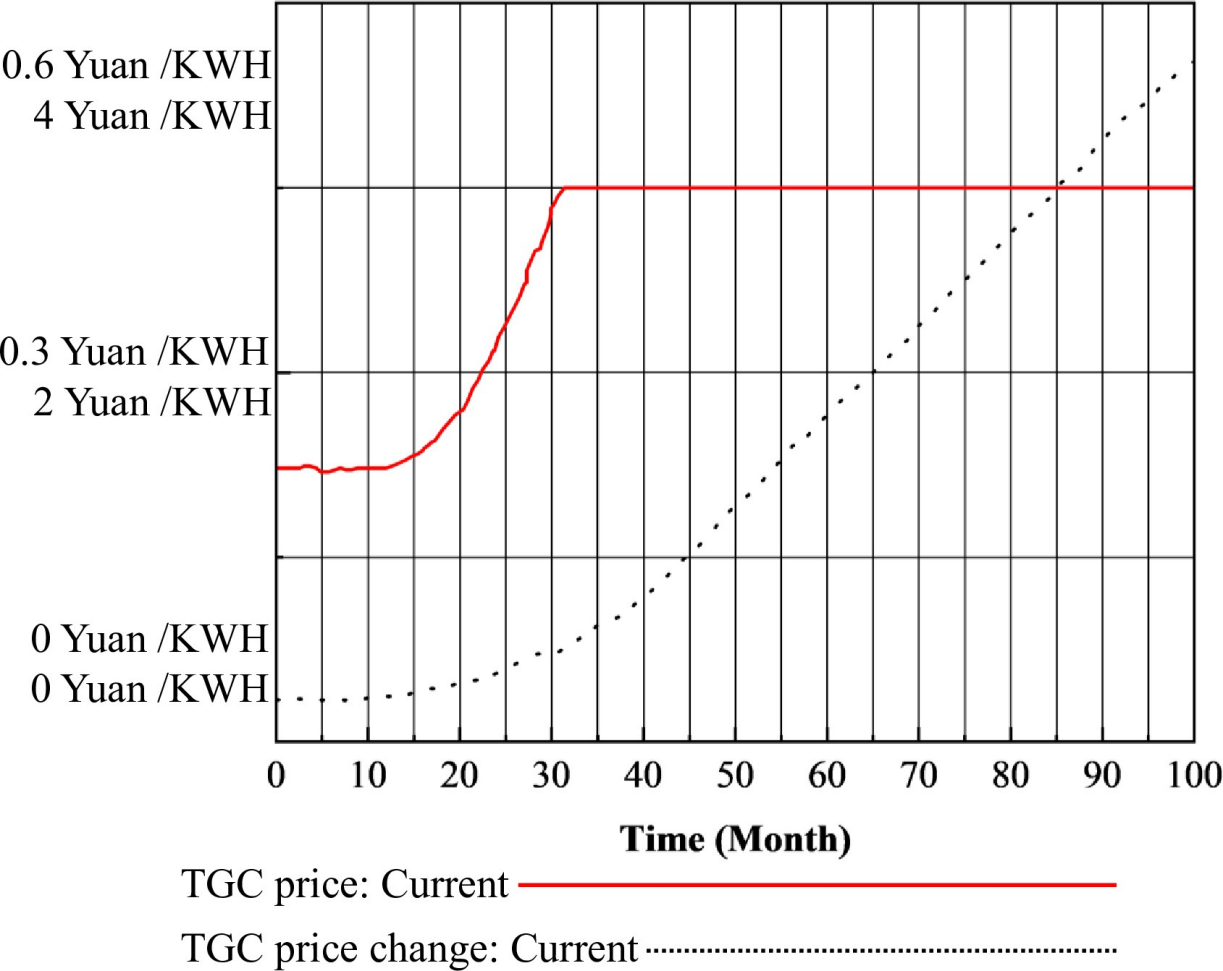
TGC prices under the three-market coupling mechanism.

The direct impact of green certificate prices on the TGC market is a negative correlation between prices and the amount of renewable energy generation. However, its role within the electricity market is multifaceted. Green certificates, which serve as subsidiary documents for green electricity, participate in electricity market transactions. The total sales price of green electricity encompasses both the market price of electricity per unit and the additional value attached to green certificates. An increase in electricity prices raises green certificate prices, consequently slowing down the installation and generation of renewable energy. This dynamic ultimately leads to an equilibrium between green certificate prices and renewable energy generation within the green certificate trading market. Based on the calculations of the SD model, the changes in TGC price are shown in Figs 8 and 9.

In Fig 8, during the first year, TGC prices remained relatively stable at the initial price of 0.22 Yuan/KWH. From December to month 37, the TGC prices gradually rose at a slow pace, reaching the capped price of 0.45 Yuan/KWH, which was maintained until month 66. In month 70, TGC prices declined to below 0.15 Yuan/KWH. Between months 71 and 95, TGC prices fluctuated, but generally remained within a narrow range around 0.20 Yuan/KWH, with predictions indicating stability in the near future. In Figs 7 and 9, it is evident that under the coupling mechanism, the TGC prices reached the upper limit of 0.45 Yuan/KWH earlier compared to the standalone TGC mechanism, and remained at this higher level for an extended period.

Regarding the divergence in the trends of TGC prices under the two mechanisms depicted in Figs 8 and 9, at the beginning of the implementation of the CT mechanism, the baseline carbon price was relatively low at 50 Yuan / tons. This meant that the cost of purchasing carbon emissions rights was lower than investing in energy saving measures or renewable energy generation. Consequently, traditional energy generation companies intensified investments in coal-fired power plants, accepting penalties, which resulted in inadequate installation of renewable energy, thus prolonging the time required for green certificate supply to match demand. This extension consequently prolonged the period during which green certificate prices remained at high levels. Late-stage fluctuations in TGC trends in Fig 8 are expected to manifest after 100 months.

**Fig 10:**
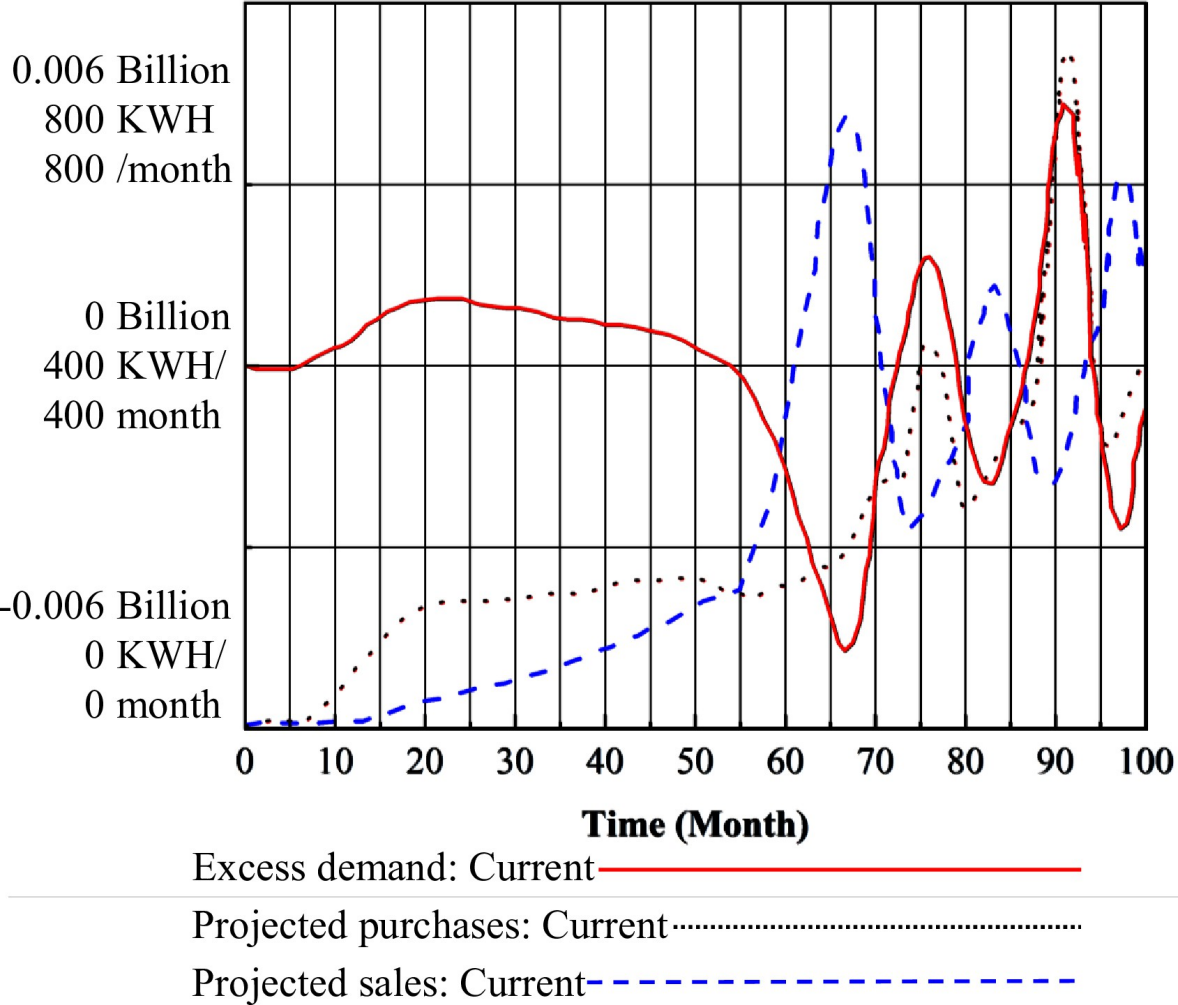
Changes in excess TGC demand and related variables.

The trends in the projected purchases of TGC by the grid, the projected sales of TGC by green power manufacturers, and the trend in the excess demand for TGC are shown in Fig 10.

The variable trends depicted in Fig 10 indicate the following: Ⅰ. For the grid, within the initial 4 years of the implementation of the mandatory market share scheme for renewable energy generation, the TGC market experienced supply-demand imbalances, leading to a rise in TGC prices to their peak. The expected purchase volume of TGC by the grid gradually increased, reaching 1800 billion KWH/month. As the supply of TGCs in the market gradually became sufficient, resulting in a decrease in TGC prices, the anticipated purchase volume of TGC in the grid increased to meet the growing quota requirements. Subsequently, over the next few years, the grid’s anticipated purchase volume fluctuated around 4000 billion KWH/month due to fluctuations in TGC prices. Ⅱ. For green electricity generators, within the initial 4 years of the implementation of the Renewable Energy Generation Mandatory Market Share Scheme, due to the continuous increase and eventual maintenance of high TGC prices, green electricity generators have heavily invested in renewable energy generation. This led to a rapid and steady increase in their anticipated sales volume, ultimately reaching around 1800 billion KWH/month. With the sales volume growth rate surpassing that of the purchase volume, a short-term equilibrium was reached at 1500 billion KWH/month by month 55. Over the subsequent years, as renewable energy installations were completed, resulting in a sudden increase in renewable energy power on the market, the supply of TGCs exceeded demand. Green power generators, driven by strong sales desires, significantly increased their anticipated sales volume, peaks at 6800 billion KWH/month. Similarly, the anticipated sales volume followed a fluctuating trend with TGC prices. Ⅲ. Regarding the TGC excess demand curve, before month 55, the anticipated purchase volume consistently exceeded the anticipated sales volume, leading to an increase in excess demand. Between months 55 and 70, the significant gap between anticipated purchase and sales volumes caused excess demand to rapidly drop to 0 KWH. Subsequently, with the increase in renewable energy power, the decrease in TGC prices, and the gradual stabilization of the TGC market, excess demand fluctuated with changes in the difference between the two volumes.

### 3.3 Coupling analysis of the electricity market

**Fig 11:**
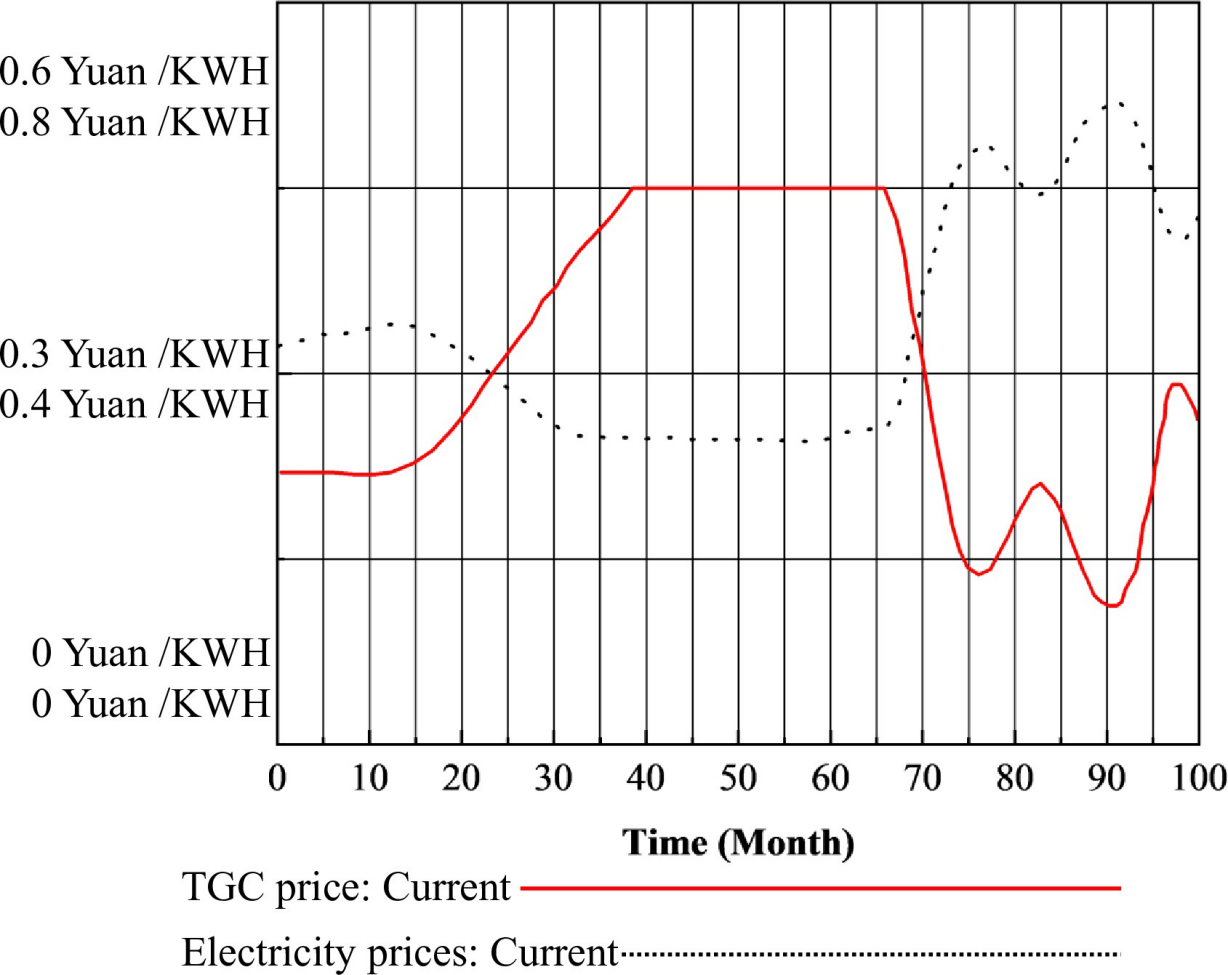
Changes in electricity price under the TGC mechanism.

The relationship between TGC prices, CT prices, and electricity prices, along with the trend of electricity prices under the coupling mechanism of the three markets, is shown in Fig 11.

In Fig 11, it is evident that the electricity price increased from an initial value of 0.43 Yuan/KWH to 0.45 Yuan/KWH during the period of 0–16 months. Subsequently, from 16 to 31 months, the electricity price decreased to a minimum of 0.33 Yuan/KWH, remaining constant at this level until the 31st month. After the 31st month, the electricity price rebounded sharply to its maximum value of 0.65 Yuan/KWH, fluctuating around 0.6 Yuan/KWH thereafter. This outcome aligns with the fundamental economic principle of a negative correlation between electricity prices and TGC prices. As subsidies obtained from the green certificate market increase, the profits derived from the electricity market diminish, thereby maintaining stability in both the green certificate and electricity markets and facilitating equilibrium through the inherent interaction of market mechanisms.

**Fig 12:**
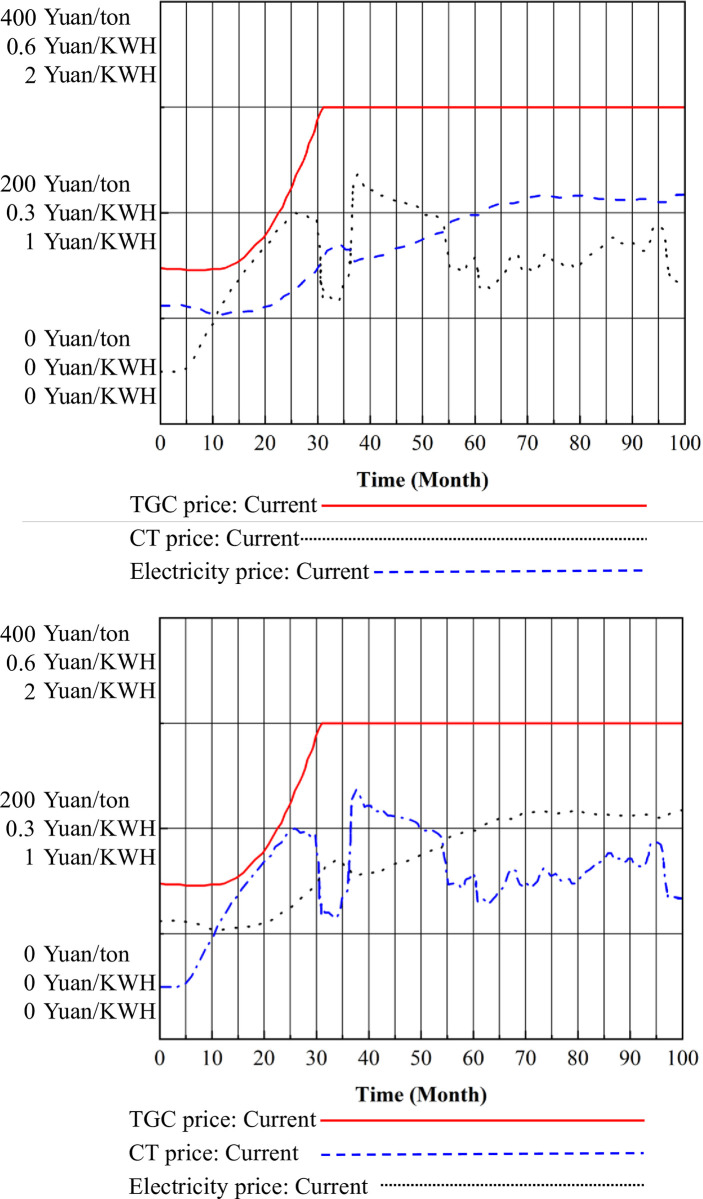
The electricity price under the three-market coupling mechanism.

Under the coupling mechanism of the three markets, the fluctuation of electricity prices in response to changes in TGC prices and CT prices is illustrated in Fig 12.

In Fig 12, it is evident that electricity prices decreased from the initial price of 0.56 to 0.52 Yuan/KWH during the period of 0–15 months. Subsequently, between months 20 and 34, electricity prices increased to 0.84 Yuan/KWH. In month 38, they decreased to 0.77 Yuan/KWH, followed by a gradual increase to 1.09 Yuan/KWH between months 40 and 100, maintaining relative stability after minor fluctuations. This trend aligns with the fundamental economic understanding of the interrelationship between TGC prices, CT prices, and electricity prices. Specifically, as TGC prices increase, electricity prices decrease, whereas an increase in CT prices leads to higher electricity prices. Ultimately, under the combined influence of the TGC and CT mechanisms, electricity prices are affected by both TGC and CT prices. The observed trend indicates that, within the current parameter settings and time frame, the impact of the CT mechanism on electricity prices outweighs that of the TGC mechanism.

## 4. Conclusions

Studying the dynamics of the electricity trading market under the influence of the CT market and the TGC market is an effective way to safeguard investors’ returns and control investment risks. In this study, CT market models, TGC market models, and multi-market coupling models were constructed on the basis of the SD model. The impact of various variables on market prices and supply-demand was calculated, and the influence trends of the CT market and the TGC market on electricity prices were analyzed. These findings can promote the prioritized development of renewable energy, reducing phenomena such as curtailment in the production process of renewable energy. The establishment of renewable energy quota systems and TGC markets is advantageous in reducing government management costs and fostering a fair and competitive market environment. Furthermore, it better reflects the external value of renewable energy electricity, provides sales channels for various types of renewable energy generation, reduces the cost of renewable energy generation, and expands the scale of renewable energy electricity production, thereby offering consumers a broader range of green energy products. The main results are as follows.

1. The system dynamics modeling approach applied in this study provides valuable insights into the complex interactions between carbon trading, green certificate trading, and electricity markets. Through the analysis of differential equations and feedback mechanisms, the study elucidates the dynamic behavior of these interconnected systems over time.
2. Quantitative results demonstrate the significant impact of market coupling mechanisms on carbon prices, green certificate prices, and electricity prices. Under the three-market coupling mechanism, carbon trading prices stabilize around 150 Yuan/ton, while green certificate prices reach a peak of 0.45 Yuan/KWH. This, in turn, influences electricity prices, which fluctuate between 0.33 and 1.09 Yuan / KWH during the simulation period.
3. These findings underscore the importance of understanding the synergistic effects among carbon emissions reduction policies, renewable energy incentives, and market dynamics. Such insights are crucial for policymakers and market stakeholders in formulating effective strategies to achieve sustainable energy transitions and mitigate climate change impacts.

Future research could investigate in more depth the long-term implications of different policy interventions and market dynamics on carbon emissions reduction targets, renewable energy deployment, and overall energy market stability. Additionally, the integration of real-world data and scenario analysis could further enhance the predictive power and applicability of SD models to guide policy decision-making and promote sustainable energy transitions.
